# Antiviral activity of interleukin-11 as a response to porcine epidemic diarrhea virus infection

**DOI:** 10.1186/s13567-019-0729-9

**Published:** 2019-12-21

**Authors:** Yuchen Li, Qingxin Wu, Yuxin Jin, Qian Yang

**Affiliations:** MOE Joint International Research Laboratory of Animal Health and Food Safety, College of Veterinary Medicine, Nanjing Agricultural University, Nanjing, Jiangsu China

## Abstract

Interleukin-11 (IL-11), a well-known anti-inflammatory factor, provides protection from intestinal epithelium damage caused by physical or chemical factors. However, little is known of the role of IL-11 during viral infections. In this study, IL-11 expression at mRNA and protein levels were found to be high in Vero cells and the jejunum of piglets during porcine epidemic diarrhea virus (PEDV) infection, while IL-11 expression was found to be positively correlated with the level of viral infection. Pretreatment with recombinant porcine IL-11 (pIL-11) was found to suppress PEDV replication in Vero E6 cells, while IL-11 knockdown promoted viral infection. Furthermore, pIL-11 was found to inhibit viral infection by preventing PEDV-mediated apoptosis of cells by activating the IL-11/STAT3 signaling pathway. Conversely, application of a STAT3 phosphorylation inhibitor significantly antagonized the anti-apoptosis function of pIL-11 and counteracted its inhibition of PEDV. Our data suggest that IL-11 is a newfound PEDV-inducible cytokine, and its production enhances the anti-apoptosis ability of epithelial cells against PEDV infection. The potential of IL-11 to be used as a novel therapeutic against devastating viral diarrhea in piglets deserves more attention and study.

## Introduction

Following a worldwide pandemic in the year 2013, porcine epidemic diarrhea virus (PEDV) has caused immense economic losses to the global swine industry [1–3]. The disease is characterized by acute viral diarrhea in swine and has a mortality rate that is as high as 90% in neonatal piglets [4]. Microscopic examination of PEDV-infected nursing pigs reveals severe necrosis and atrophic villi of the small intestinal enterocytes [5]. Due to the special features of the porcine intestinal mucosal immune system, traditional vaccination methods do not provide effective protection against gastroenteric pathogens. Insufficient immunological protection against PEDV is mainly due to the following: (1) serum antibodies induced by intramuscular immunity that has no effect on intestinal mucosal pathogens; (2) antigens inoculated by oral immunization that are easily degraded in the digestive tract and often only induce low local immune efficiency [6, 7]. Therefore, strategies for improving the function of the intestinal mucosal immune system, including physical barriers, specific sIgA and antiviral cytokines, deserve increased attention for developing PEDV therapeutic targets and prevention.

As a member of the IL-6 cytokine family, interleukin-11 (IL-11) is secreted by a broad range of cell types, including hepatocytes, gastrointestinal epithelial cells, T cells, B cells and macrophages [8, 9]. The binding of IL-11 to its alpha receptor, IL-11Rα results in a dimeric complex that interacts with GP130 to form a tetrameric complex [10–12]. This reaction further activates phosphoinositide 3-kinase (PI3K), mitogen-activated protein kinase (MAPK) and the signal transducer and activator of transcription (STAT) [13, 14]. Low levels of IL-11 mRNA are found throughout the body but are rarely detected in tissues of healthy individuals [15]. However, in many inflammatory diseases, IL-11 is readily detectable in tissues and plays an important anti-inflammatory function. Due to its strong ability in promoting proliferation and suppressing apoptosis of enterocytes, IL-11 plays an important protective role against multiple types of IEC damage. Studies have shown that recombinant human IL-11 can reduce the incidence of extensive necrotizing enterocolitis (NEC) in infants [16]. Further studies reveal that IL-11 can induce an anti-apoptotic effect and the proliferation of intestinal epithelial cells (IECs) and protect against intestinal damage caused by neutron and X-ray irradiation injury in mice [17, 18]. Moreover, IL-11 can alleviate intestinal inflammation in mice suffering from inflammatory bowel disease by inhibiting the production of many inflammatory cytokines, such as TNF-a, IL-1β and IL-6 [19].

Many viruses have the ability to actively induce apoptosis as a response to viral replication, thereby facilitating the release and dissemination of viral progeny into neighboring cells [20]. This pro-apoptotic effect plays a pathogenic role that contributes to PEDV-induced cell damage, tissue injury and disease severity in piglets. Previous studies have shown that PEDV can induce apoptotic cell death via an AIF-mediated pathway that plays a critical role in promoting PEDV replication and pathogenesis [21, 22]. We hypothesize that the antiapoptotic approach may be one of the most appropriate strategies to combat PED.

Given the critical role of IL-11 in epithelial regeneration, necrosis and apoptosis reduction, as well as inflammatory inhibition, we illustrate the potential function of IL-11 in PEDV infection. Our findings identify the antiviral function of cytokine IL-11 during PEDV infection and suggest that IL-11 may be a potential novel antiviral therapeutic target.

## Materials and methods

### Cells and reagents

African green monkey kidney Vero E6 cells were grown and maintained in DMEM supplemented with 10% FBS and incubated at 37 °C with 5% CO_2_. The PI3K-specific inhibitor LY294002, STAT3-specific inhibitor S3I-201 and Akt inhibitor MK-2206 2HCl were purchased from Selleck Chemicals (Houston, USA), and were diluted to 10 mM in DMSO.

### Virus and infection

The wild-type PEDV strain Zhejiang08 was preserved by our laboratory, which was clustered with the emerging virulent strain [23]. Vero E6 cells were inoculated with the virus at a multiplicity of infection (MOI) of 0.1 and cultured in serum-free DMEM for 1 h at 37 °C with 5% CO_2_. The inoculum and unattached virus were removed and fresh growth medium was added. Infected cells were analyzed at the indicated time.

The piglets used in this experiment were seronegative for antibodies against PEDV, Porcine reproductive and respiratory syndrome virus (PRRSV), Transmissible gastroenteritis virus (TGEV) and Porcine circovirus type 2 (PCV2). Fifteen 3-day-old nursing piglets were randomly assigned to two groups and housed separately: PEDV infected (*n* = 12) and mock (*n* = 3). Piglets of the PEDV infected group were challenged with 1 mL PEDV (10^7^ PFU/mL) by oral inoculation. In the mock infected group, the same volume of DMEM medium was inoculated as a negative control. The animals were artificially fed with milk every 3 h throughout the experiment. After PEDV inoculation, pigs were monitored for clinical signs 2–3 times daily until diarrhea. When piglets exhibited classical PEDV symptoms, the diarrhea piglets were necropsied, sampled and detected for PEDV infection. Meanwhile, three piglets from the PEDV infected group were euthanatized at 12 h, 24 h and 48 h after virus inoculation, and jejunal tissues of the piglets were collected. The animal studies were approved by the Institutional Animal Care and Use Committee of Nanjing Agricultural University and followed National Institutes of Health guidelines for the performance of animal experiments.

### Phylogenetic analysis and prokaryotic expression of pIL-11

An alignment of porcine IL-11 (pIL-11) sequence with other mammalian IL-11 molecules was carried out using the MegAlign program of DNAstar (DNAstar Inc., Madison, USA). A phylogenetic tree based on the amino acid sequence were constructed with the computer program MEGA version 5.0, utilizing the neighbor-joining method. The sequence data for phylogenetic analysis were taken from the GenBank nucleotide sequence database with the following accession numbers: pig IL-11, XP_020950667.1; human IL-11, NP_000632.1; mouse IL-11, NP_032376.1; monkey IL-11, XP_007996343.1; bovine IL-11, XP_024835139.1; goat IL-11, XP_024835139.1 and chicken IL-11, XP_024998644.1.

To clone pIL-11, total cellular mRNA was isolated from the small intestine of the PEDV infection pig, and cDNA was synthesized using HiScript II Q RT SuperMix for qPCR (Vazyme, China) according to the manufacturer’s instructions. Primers used for IL-11 are summarized in Table 1. Amplification was performed using PrimeSTAR GXL DNA polymerase (Takara, Dalian, China). The cDNA fragment encoding the pIL-11 was inserted into vector pET-32a vector by T4 DNA ligase (Thermo Scientific) to generate the recombinant plasmid pET-32a-pIL-11. Finally, pET-32a-pIL-11 was transformed into BL21 (DE3), while the recombinant mpIL-11 was purified by a Ni–NTA column and removed Trx-tags by Enterokinase (EK) (Sangon Biotech, Shanghai, China).

**Table 1:** **Primers used for qPCR**

Genes	Primers	Sequence (5′–3′)*	Accession
GAPDH	Forward	TCATCATCTCTGCCCCTTCT	NM_001206359.1
	Reverse	GTCATGAGTCCCTCCACGAT	
PEDV-M	Forward	ATGCATGGGCTAGCTTCCAG	JX002693.1
	Reverse	GTAGTGAGAAGCGCGTCAGT	
IL-11(Pig) (PCR)	Forward	GTGGTGGTGCTCGAGCAGCCGAGTCTT	XM_021095008.1
	Reverse	GCTGATATCGGATCCATGAACAGTGTTT	
IL-11(Pig) (qPCR)	Forward	CCGCACAGCTGAGAGACAAAT	XM_021095008.1
	Reverse	GCCTCAGGTAGGAAAACAGGT	
IL-11 (Green monkey)	Forward	CCCGAGTGTGCTGACAAGG	XM_007998152.1
	Reverse	CCTGAAGACCCTGGAGCCTG	
Bcl-2	Forward	ATGTGTGTGGAGAGCGTCAA	XM_008013867.1
	Reverse	GGGCCGTACAGTTCCACAAA	
Bcl-W	Forward	GCAGGTATTGGTGAGTCGGA	XM_008020066.1
	Reverse	ATAAACCTTGCACCTCTCCCAG	
MCL-1	Forward	GCAGGTATTGGTGAGTCGGA	XM_008019267.1
	Reverse	GCTCTGGAGACCTTACGACG	

### Cell cytotoxicity assay

The activity of Vero E6 cells was assayed using the Cell Counting Kit-8 (CCK-8) method. Vero E6 cells were plated at a density of 4 × 10^5^ cells/mL in 96-well plates at 37 °C and treated with different levels of purified pIL-11 or inhibitors for the indicated time, followed by treatment with 10 μL/well CCK8 (5 mg/mL). The cells were incubated for an additional 2 h. The OD value was read at 570 nm on a microplate reader. The cell viability (%) was calculated as the percent ratio of absorbance of the samples against the non-treated control medium.

### Assessment of apoptosis by Annexin V and PI staining

To evaluate the protective effect of pIL-11 on the PEDV-induced apoptosis, Vero E6 cell monolayer was pretreated with IL-11 for 18 h and then infected with PEDV at a MOI of 0.1 for another 36 h. Cells were harvested and labeled with an anti-Annexin V-FITC Apoptosis Detection Kit (Vazyme, China). The fluorescent signals of Annexin V and PI were detected at channels FL-1 and FL-3, respectively, and were analyzed by flow cytometry (BD FACSVerse™; BD Bioscience). All flow cytometric data were analyzed using FlowJo software. Cells negative for PI uptake and positive for Annexin V were considered apoptotic.

### Target-specific silenced cell generation

shRNA targeting sequences against IL-11 (XM_007998152.1) were designed using online design tools BLOCK-It RNAi Designer (Life Technologies), and three short target sequences with the best scores were selected. Targeting sequences are described in Additional file 1. The shRNA were cloned into the pLVX-shRNA1 vector (Takara, Dalian, China) containing EcoRI and BamHI sites. Lentiviral were produced in 293T cells following a standard liposome transfection with three packaging plasmids [24]. Then cells were incubated with the lentiviral particles (MOI 1) in the presence of 8 μg/mL Polybrene (Sigma-Aldrich). Forty-eight hours after infection, the cells were incubated with 6 μg/mL puromycin for selection. IL-11 target-specific silenced cell lines and the negative control (NC) scrambled vector-infected cells were named shRNA-IL-11 knock know (IL-11KD) and shRNA-negative control (shRNA-NC), respectively.

### ELISA assay for the cytokines

The medium from the cell cultures was collected, pooled, and stored in aliquots at −70 °C until analysis. The cell supernatants were measured for IL-11 levels using an IL-11 ELISA kit (R&D Systems, Minneapolis, MN, USA) according to the manufacturer’s instructions.

### Western blotting analysis

Cells were lysed in RIPA buffer containing a protease inhibitor cocktail (Thermo Scientific). The proteins were separated on 10% SDS-PAGE and transferred to polyvinylidene fluoride (PVDF) membranes (Millipore, Bedford, MA, USA). Antibodies against total STAT3, p-STAT3 (S727), p-STAT3 (Y705), total Akt, phospho-Akt (Ser473), total ERK, ERK1/2 (Thr202/Tyr204) were purchased from Cell Signaling Technology (Beverly, MA, USA). Monoclonal antibodies against IL-11 and GAPDH were purchased from Proteintech (Wuhan, China) and Beyotime Institute of Biotechnology (Shanghai, China), respectively. Monoclonal antibodies against PEDV N was purchased from Medgene labs (USA). After washing three times with TBST, membranes were exposed to species specific horseradish peroxidase (HRP)-conjugated secondary antibodies (Vazyme, Nanjing, China) followed by enhanced chemiluminescence (ECL, Thermo Scientific) detection by autoradiography. Western blotting was quantified by Quantity One (Quantity One 1-D Analysis Software 170-9600, Bio-Rad). The intensity of the bands in terms of density was measured and normalized against GAPDH expression.

### Quantitative RT-PCR

For quantitative reverse transcription-polymerase chain reaction (qPCR), total cellular RNA was extracted with TRIZOL (Life Technologies) and cDNA was synthesized with a reverse transcriptase kit (TaKaRa, Dalian, China). qPCR was performed using the Real-Time PCR system (ABI 7500, Life Technologies, USA). Gene expression was calculated with the comparative Ct method and normalized to the endogenous levels of GAPDH. The gene expression of IL-11 and PEDV was determined and analyzed by the double standard curve method. PCR products were cloned into the pJET1.2 vector (Thermo Fisher Scientific). Plasmids were serially diluted and used as standards for quantitative analysis. The initial copy number of IL-11 gene, PEDV M gene and GAPDH were calculated using the formula given in Additional file 2. The expression of apoptosis-related genes were normalized against the GAPDH expression level and are expressed as fold differences between control and treated cells according to the 2^−△△^CT method. All primer sequences used for qPCR were listed in Table 1.

### Statistical analysis

Data are presented as mean ± SD. Statistical analysis was performed using SPSS 18.0. Significance was determined by One-way analysis of variance (ANOVA). *P < 0.05, **P < 0.01. Data were combined from at least three independent experiments unless otherwise stated.

## Results

### IL-11 expression is elevated in PEDV infected host cells

**Figure 1:**
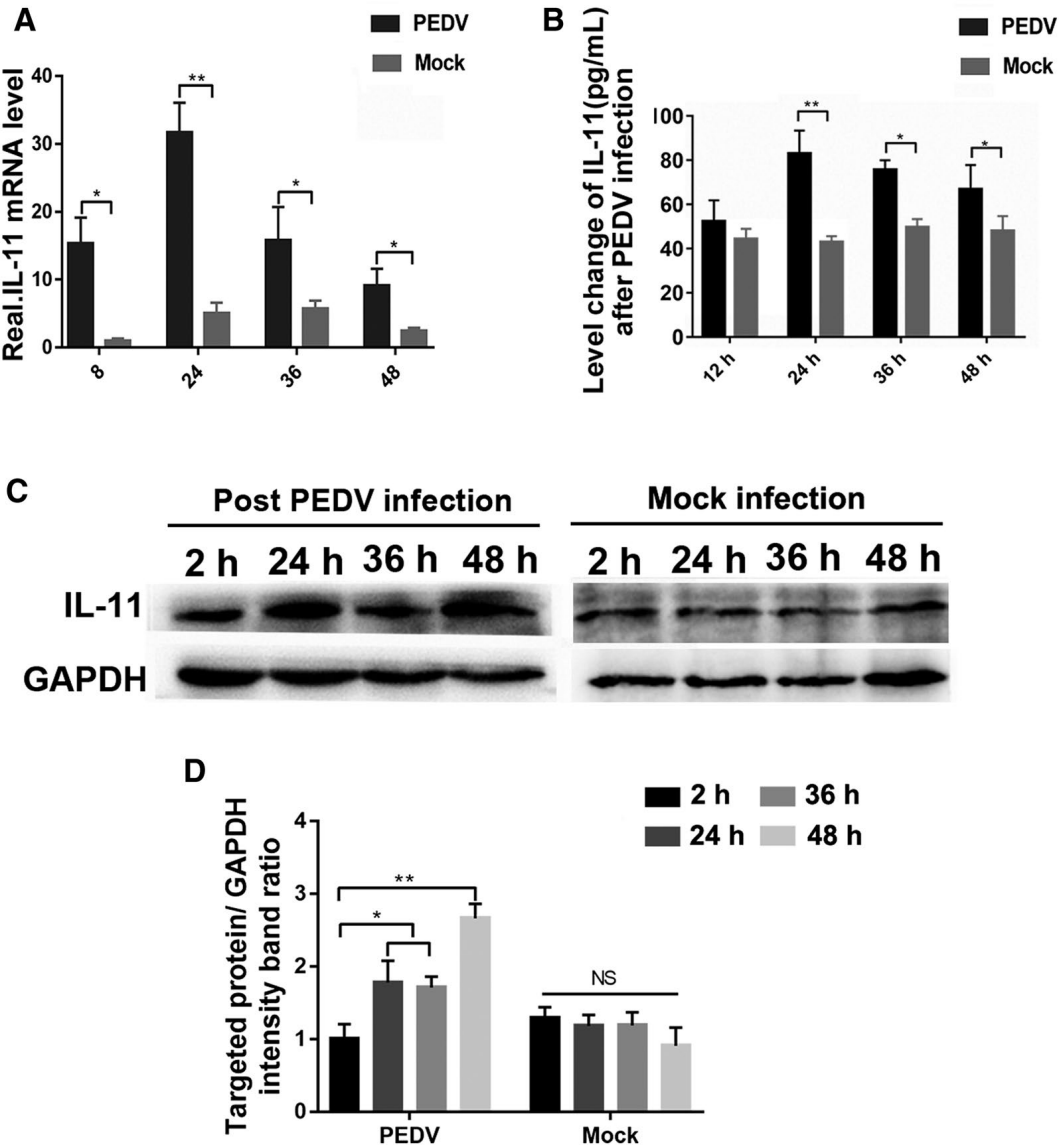
**Induction of IL-11 by PEDV in Vero E6 cells.** Vero E6 cells were mock-infected or PEDV-infected (MOI 0.1) at the time indicated, then cellular total RNA and the supernatant were harvested. **A** RNA expression level of IL-11 in Vero E6 cells quantified using qPCR. **B** The protein expression of IL-11 in the supernatant media determined using ELISA. **C** The protein expression on IL-11 in Vero E6 cells determined using Western blotting. **D** Statistical results of Western blotting. Data are presented as the mean ± SD of three independent experiments. *P < 0.05, **P < 0.01.

In order to investigate the induction of IL-11 gene transcription during PEDV infection, total RNA from PEDV-infected Vero E6 cells were extracted at the time indicated. IL-11 mRNA levels in PEDV-infected cells were at least sixfold higher compared with that of the controls, which increases along with viral infection, with RNA expression reaching a peak after 24 h and then gradually decreasing (Figure 1A). The protein levels of IL-11 in the culture supernatant were also detected using ELISA. Based on the IL-11 mRNA level in PEDV infected cells, the quantity of secreted IL-11 in supernatants show a similar increasing trend after viral infection (Figure 1B). However, the kinetics profile of intracellular IL-11 protein detected by Western-blot was different from that of mature IL-11 in supernatants. Many IL-11 accumulate in cells at 48 h post-infection (hpi), which implies that the release of mature IL-11 may be influenced by PEDV infection (Figures 1C and D). These results demonstrate that infection of Vero E6 cells with PEDV stimulates the production of IL-11.

### IL-11 expression is highly correlated with PEDV infection in piglets

**Figure 2:**
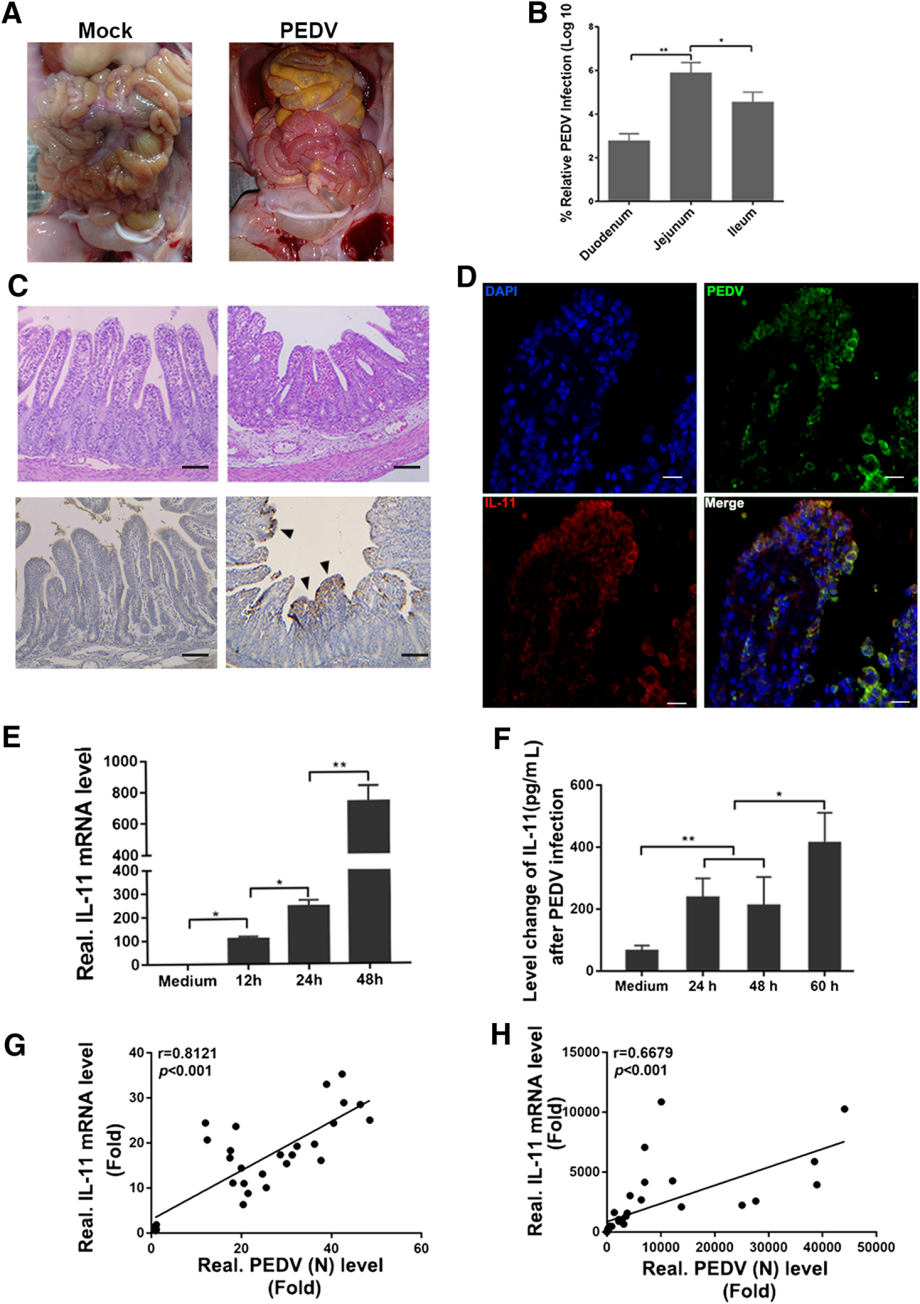
**Quantity of IL-11 in PEDV-infected piglets.** Three-day-old piglets were inoculated orally with PEDV and sacrificed 60 h after challenge. **A** The necropsy results exhibit gross lesions of the intestine in piglets after PEDV oral inoculation. **B** Viral RNA expression in different intestine segments of the diarrheic piglets. **C** Pathological examination and immunohistochemical staining for the detection of PEDV antigen (N protein) in the jejunum of PEDV-inoculated piglets. The black arrow indicates PEDV antigen-positive cells. Bars, 20 μm. **D** Immunofluorescence staining of PEDV antigen (N protein) and IL-11 in the jejunum of the PEDV inoculated piglets. Blue, DAPI; green, PEDV; red, IL-11. Bars, 25 μm. Moreover, three piglets from PEDV infected group were euthanized at 12 h, 24 h and 48 h after virus inoculation, and jejunal tissues of piglets were collected. The jejunal tissues from mock infected piglets were also acquired as a negative control. **E** IL-11 mRNA levels in the jejunum was quantified using qPCR. Medium: jejunal tissues from mock infected piglets. **F** IL-11 secreted from intestinal wash was detected using ELISA. **G** Correlation analysis between PEDV mRNA and IL-11 mRNA levels in PEDV-infected Vero E6 cells. **H** Correlation analysis between PEDV mRNA and IL-11 mRNA levels in PEDV-infected jejunum tissues. Linear growth trend (solid line); correlation coefficient (r); Pearson correlation analysis was used to determine r values; Student t test was used to determine P values. Data are presented as mean results ± SD from three independent experiments. *P < 0.05, **P < 0.01.

In order to validate the reality of this phenomenon in piglets, changes in IL-11 expression in response to PEDV infection were also examined. After PEDV challenge, the clinical signs included severe watery diarrhea with vomiting detected in oral PEDV-inoculated piglets at 54 hpi. Then, the piglets were anesthetized with pentobarbital sodium (100 mg/kg) and sacrificed for macroscopic examination at 60 hpi. The piglets were found to exhibit moderately thin and transparent intestinal walls in the small intestine, with an accumulation of large amounts of fluid in the intestinal lumen (Figure 2A). PEDV mainly colonizes the jejunum and its viral titer is significantly higher than that of the duodenum and ileum (Figure 2B). Histopathological results confirm hyperemia and multifocal diffuse villous atrophy in the jejunum (Figure 2C). Immunohistochemical analysis further shows that a large number of PEDV-positive cells are found in the jejunum of piglets with diarrhea, while PEDV antigens were mainly observed in the cytoplasm of villus epithelial cells (Figure 2C). Meanwhile, the IL-11 secreted by PEDV infected cells in the jejunum of the infected piglets were also observed in Figure 2D.

Jejunal tissue from PEDV-infected piglets were collected at the times indicated and analyzed. IL-11 expression at both mRNA and protein level increase in the intestine during the course of PEDV infection (Figures 2E and F). In order to verify whether IL-11 expression is related with viral infection, PEDV M and IL-11 gene copies in Vero E6 cells (Figure 2G) and intestinal tissues (Figure 2H) were compared using the Pearson correlation. A statistically significant correlation was observed between PEDV and IL-11 mRNA levels not only in vitro, but also in vivo. These results indicate that IL-11 expression is positively correlated with PEDV infection.

### Cloning and expression of pIL-11

**Figure 3:**
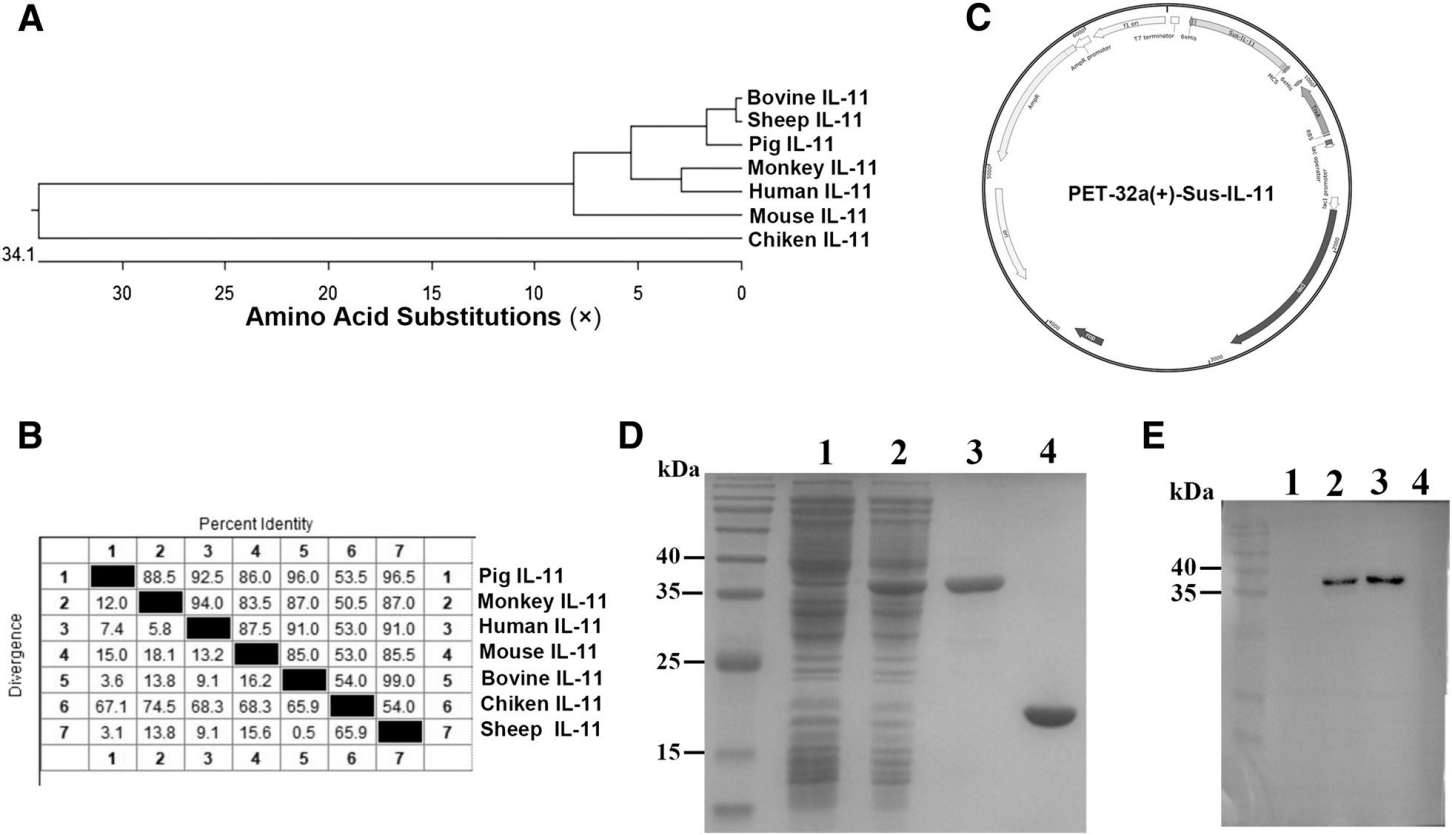
**Prokaryotic expression of mpIL-11. A** Phylogenetic analysis and **B** amino acid (aa) sequence homologies of pIL-11 in comparison with other mammalian IL-11 molecules (monkey, human, mouse, bovine, chicken and sheep). Phylogenetic analysis based on the aa sequence of the IL-11 was constructed using MEGA version 5.0 software, utilizing the neighbor-joining method and bootstrapping for 1000 replicates with a value > 70%, in order to determine the percentage reliability of each internal node. The amino acid (aa) sequence homologies among the species were analyzed using the MegAlign program of DNAstar (DNAstar Inc.). **C** The protein mpIL-11 containing 600 bp was amplified specifically from mRNA of pig intestines and cloned into *E. coli*-expressing vector pET-32a. **D** SDS-PAGE analysis of the expression and purification of mpIL-11 from *E. coli* BL21(DE3)/pET-32a. 1: total protein of empty pET-30a vector; 2: protein supernatant including His-Trx-pIL-11; 3: affinity-purified His-Trx-pIL-11 protein; 4: unlabeled pIL-11 protein after enterokinase digestion. **E** A similar protein sample order to that of D was determined through Western blotting using a monoclonal antibody against His protein.

Growing evidence indicates that IL-11 can play an important role in protecting intestinal epithelial cells against multiple types of cell damage. However, the function of IL-11 during viral infection has not been previously reported. Therefore, we sought to determine the biological function of IL-11 during viral infection. Phylogenetic analyses based on its amino acid sequence reveals that pIL-11 can be grouped with other mammalian IL-11 of humans, monkeys, mice, bovines and sheep, with a high identity score (range from 86 to 96.5%) (Figures 3A and B).

The full length of pIL-11 of 600 bp, was successfully amplified using pig intestinal cDNA. Then, pIL-11 was cloned into an *E. coli*-expressing vector, pET-32a, to construct a fusion expression plasmid (Figure 3C). The fusion protein His-Trx-pIL-11 with an expected size of approximately 39 kD was found to be expressed in a soluble form, purified using Ni^2+^-chelating chromatography, and cleaved by Enterokinase (EK) at 37 °C for 4 h to release recombinant pIL-11 at approximately 21 kD (Figure 3D). The expression of His-Trx-pIL-11 was further confirmed using Western blotting with mouse anti-his monoclonal antibodies, while EK digested recombinant pIL-11 did not show specific immunoreactivity (Figure 3E).

### IL-11 hampers PEDV infection in host cells

**Figure 4:**
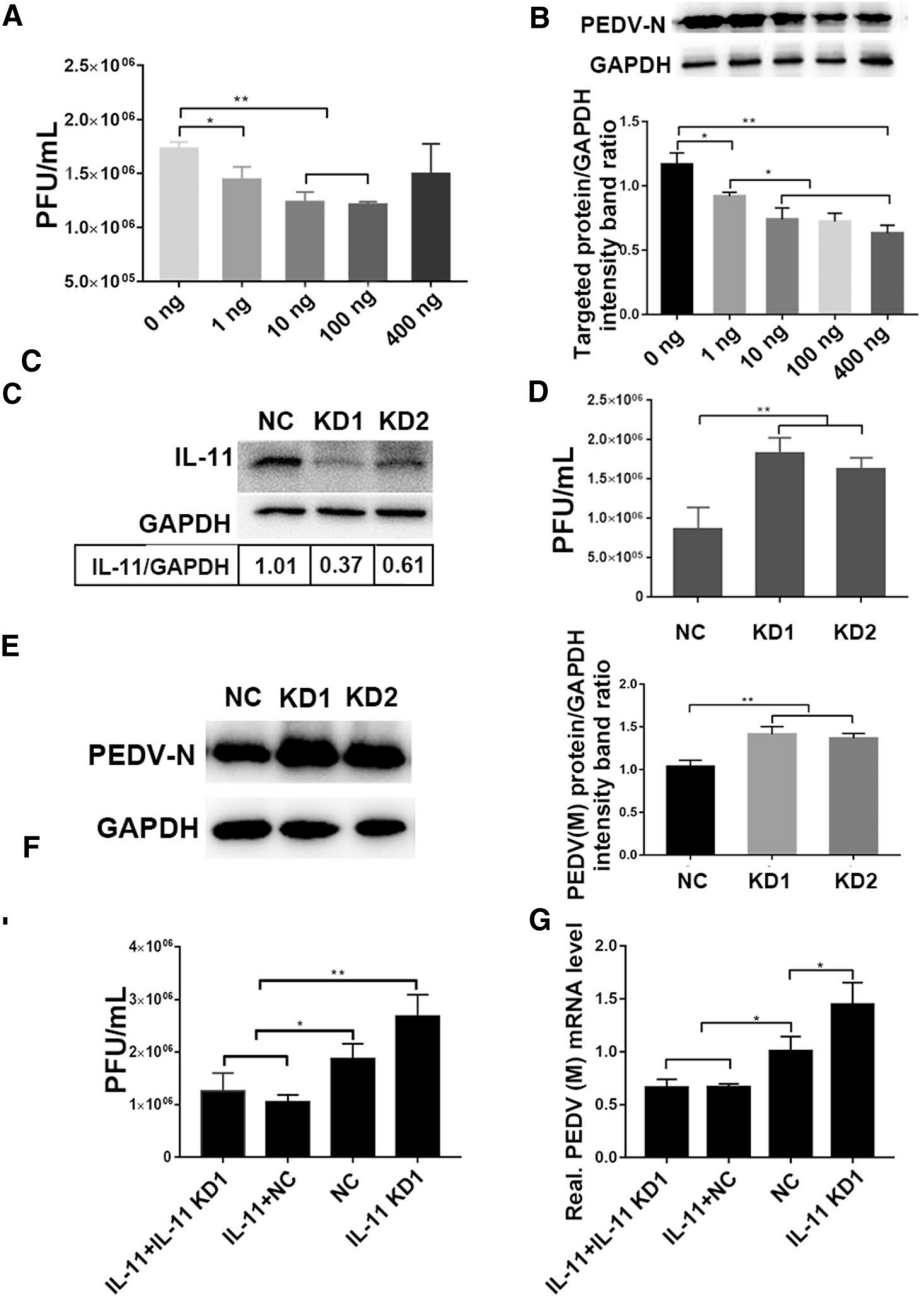
**Influence of mpIL-11 on PEDV replication.** Vero E6 cells were inoculated with PEDV at a MOI of 0.1, followed by pIL-11 (range from 1 to 400 ng) stimulation for 18 h. **A** Virus titers in the culture supernatant were measured using plaque assay. **B** Infection of Vero E6 cells with PEDV at 24 hpi was confirmed using Western blotting. **C** IL-11 shRNA verification. The IL-11 targeting shRNA significantly inhibited IL-11 expression in Vero E6 cells, which was verified using Western blotting. **D**, **E** IL-11 knockdown enhances PEDV infection. Vero E6 cells were transfected with shRNA targeting IL-11 or control (scrambled) shRNA, following infection with PEDV. Viral infection results at 24 hpi are presented. NC, KD1 and KD2 represent Vero E6 cells transfected with shRNA-negative control, shIL-11-1 and shIL-11-2, respectively. **D** The extracellular virus titers were measured using plague assay. **E** The expression of PEDV-N protein was analyzed through Western blotting using specific antibodies, as described in the Materials and methods section. **F**, **G** Vero E6 cells expressing either shIL-11-1 or control shRNA were treated with pIL-11, followed by infection with PEDV (MOI 0.1) for 24 h. **E** Viral RNA level and **F** titers of different groups were detected by qPCR and plaque assay. Data are presented as the mean ± SD of three independent experiments. *P < 0.05, **P < 0.01.

In order to test whether pIL-11 influences PEDV infection, we treated Vero E6 cells with different concentrations of purified pIL-11 for 18 h prior to PEDV infection. Plaque formation testing found that pretreatment with pIL-11 significantly decreases PEDV titers from 1 ng/mL in a dose-dependent manner (Figure 4A). pIL-11 at concentrations from 10 to 100 ng/mL is able to exhibit an excellent PEDV inhibition effect and results in 50% reduction in PEDV infection, whereas 400 ng/mL mpIL-11 results in no antiviral effect (Figure 4A). Viral protein levels were found to be consistent with the results of viral titers, which decreases to more than 40% in IL-11-treated cells (> 10 ng/mL) in comparison with that of control cells (Figure 4B). However, viral protein levels were found to have significantly decreased after 400 ng/mL of mpIL-11 treatment.

In order to further verify the role of IL-11 in PEDV infection, we generated Vero E6 cells with stably knocked down IL-11 expression using shRNA knockdown vectors. Western blotting (Figure 4C) and ELISA analyses (Additional file 3) were performed to assess IL-11 knockdown efficiency. As shown in Figure 4C, Vero E6 cells transfected with shIL-11-1 and shIL-11-2 exhibit significantly decreased levels of IL-11, compared with cells transfected with shRNA-NC, confirming the knockdown of IL-11 in these cells. Moreover, suppression of IL-11 expression increases viral progeny yield and protein expression in PEDV infected Vero E6 cells, compared with shRNA-NC transfection (Figures 4D and E). Pretreatment with pIL-11 was able to block enhanced PEDV infection in shIL-11-1 transfected Vero E6 cells (Figures 4F and G). However, IL-11 pretreatment and knockdown did not have a significant influence on the proliferation of Vero E6 cells, as shown in the results of proliferation assays (Additional file 4). These results indicate that IL-11 plays an important role in restricting PEDV infection in host cells.

### IL-11 inhibits PEDV infection via the IL-11/IL-11R/STAT3 signaling pathway

**Figure 5:**
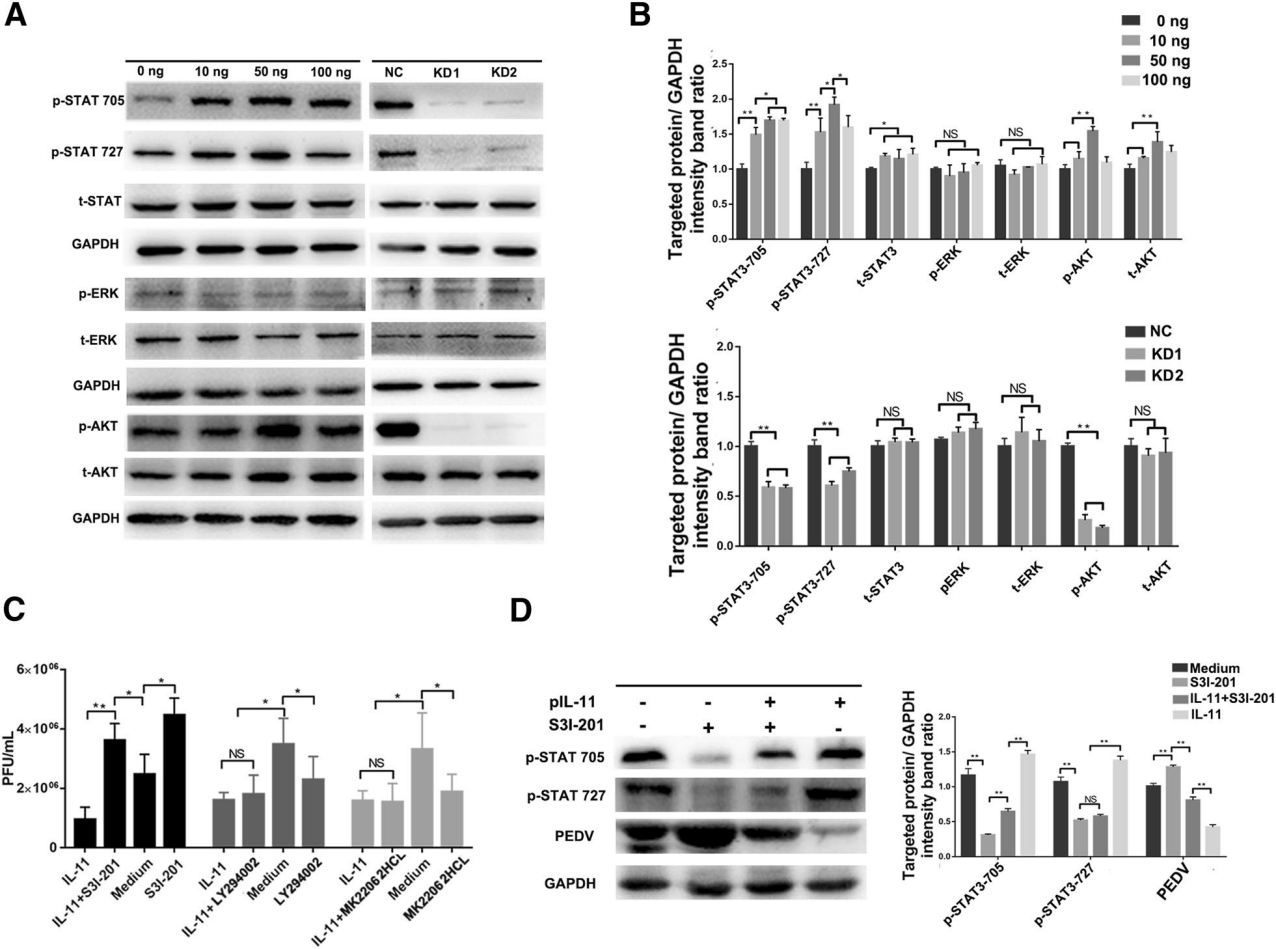
**Suppression of PEDV replication through activation of the STAT3 signaling pathway following pIL-11 treatment. A** pIL-11 induces the phosphorylation of STAT3 and AKT in Vero E6 cells. Western blotting detected specific signaling pathway makers downstream of pIL-11 after treatment with increasing concentrations of pIL-11 (range from 10 to 100 ng/mL) and in Vero E6 cells expressing either IL-11 shRNA or NC. GAPDH was used as the loading control. **B** Statistical results of the target protein expression. **C** Inhibition of STAT3 phosphorylation abrogated the anti-PEDV activity of pIL-11. Vero E6 cells were stimulated with 50 ng/mL pIL-11 for 18 h and treated with a STAT3 phosphorylation inhibitor S3I-201 (20 μM), for 24 h, PI3K inhibitor LY294002 (20 μM) and AKT phosphorylation inhibitor MK-2206 2HCl (10 μM), for 2 h. Then, Vero E6 cells were infected with PEDV for 24 h, while viral titers from different groups were detected using plaque assay. **D** Western blotting detected the protein expression of PEDV and IL-11/STAT3 pathway markers in Vero E6 cells after pretreatment with S3I-201 or 50 ng/mL pIL-11. Data are presented as the mean ± SD of three independent experiments. *P < 0.05, **P < 0.01.

Activation of multiple signaling pathways emerge when IL-11 binds to its alpha receptor. In order to determine the signaling pathways that are potentially involved in the PEDV inhibition effect induced by IL-11, we treated Vero E6 cells with different concentrations of pIL-11 (ranging from 10 to 100 ng/mL) and then analyzed the activation of Akt, STAT3 and ERK using Western blotting. As shown in Figures 5A and B, pIL-11 induces both Akt (Ser 473) and STAT3 (Ser727 and Tyr705) phosphorylation in Vero E6 cells in a dose-dependent manner, whereas Akt and STAT3 phosphorylation are inhibited in IL-11KD1 and IL-11KD2 cells. However, neither pIL-11 treatment nor IL-11 knockdown was able to cause an obvious change in the phosphorylation levels of ERK in the present study (Figures 5A and B).

In order to determine the signal pathway activation that is specifically associated with PEDV inhibition by pIL-11, three signaling pathway inhibitors, including S3I-201 (STAT3 specific inhibitor), LY294002 (PI3K specific inhibitor) and MK2206 (AKT specific inhibitor) were applied after IL-11 stimulation. The effect of these inhibitors on cell viability are shown in Additional file 5. In comparison with PI3K/AKT inhibitors, S3I-201 treatment was able to clearly promote PEDV infection and abolish the viral inhibitory effect induced by pIL-11 stimulation (Figure 5C). Moreover, the Western blotting results further indicate that treatment with S3I-201 significantly interferes with STAT3 phosphorylation and abrogates antiviral activity induced by pIL-11 stimulation (Figure 5D). This effect cannot be due to the cellular cytotoxicity caused by S3I-201, since no changes in cellular viability were observed following treatment with 20 mM S3I-201, as indicated in Additional file 5. These results indicate that IL-11 may inhibit PEDV infection by activating the IL-11/IL-11R/STAT3 signaling pathway.

### IL-11 regulates the apoptosis of host cells after PEDV infection

**Figure 6:**
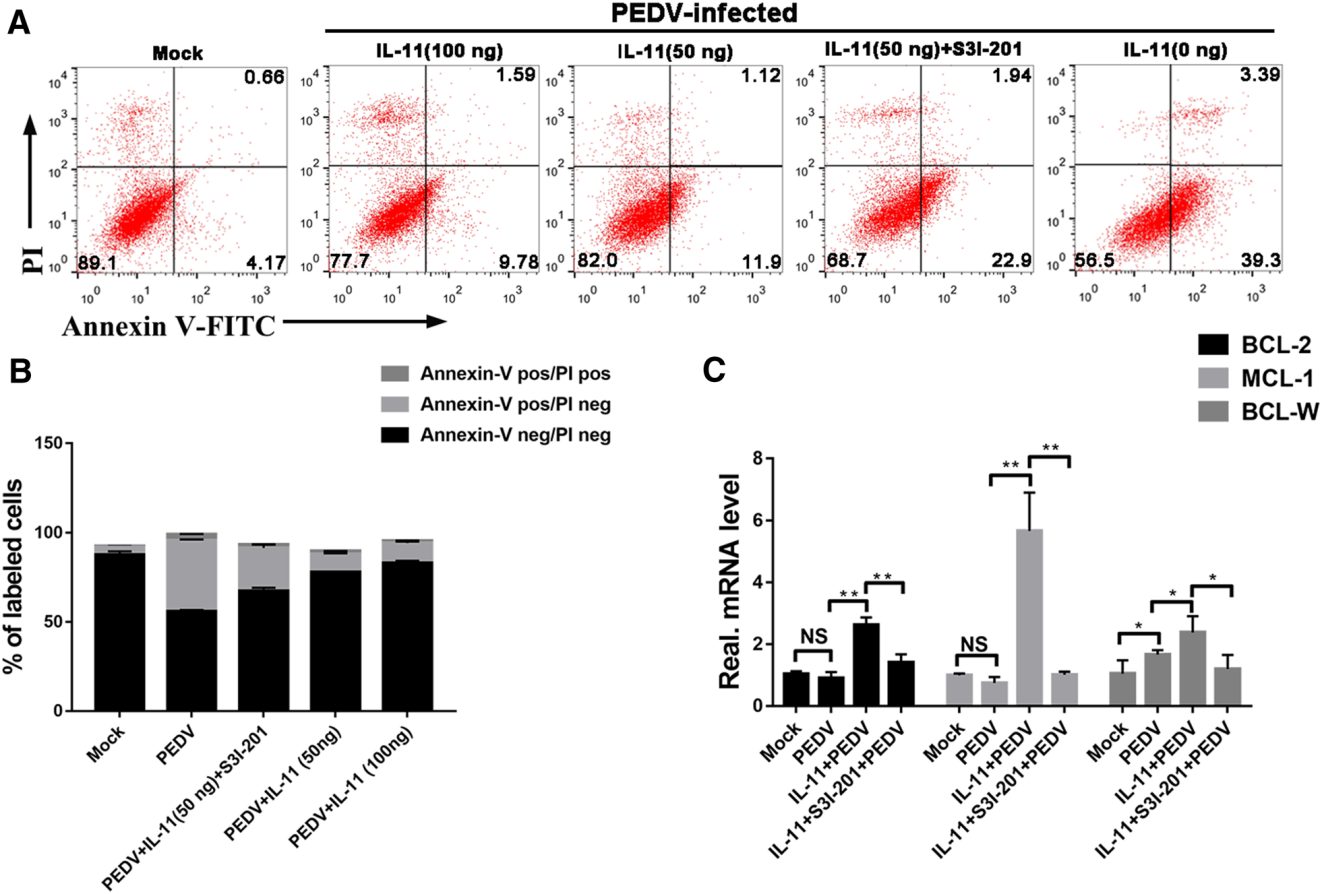
**Role of the IL-11/STAT3 signaling pathway in alleviating PEDV-induced apoptosis. A** pIL-11 reduced the apoptosis rate of Vero E6 cells after PEDV infection. Vero E6 cells were pretreated with pIL-11 and STAT3 inhibitor, and then mock-infected or infected with PEDV. Cells were harvested at 36 hpi, dually labeled with Annexin V and PI, and subjected to FACS analysis. Images are representative of three independent experiments. **B** The graph represents the percentage of each quadrant in **A**. **C** pIL-11 influenced the transcription of apoptosis-related genes after PEDV infection. qPCR assay of the mRNA levels of apoptosis-resistance genes in Vero E6 cells from different treatment groups. Amplified products were quantitated using the comparative threshold cycle (CT) method, and the results were normalized to the endogenous levels of glyceraldehyde-3-phosphate dehydrogenase (GAPDH). All results are representative of three independent experiments. Data are presented as the mean ± SD of three independent experiments. *P < 0.05, **P < 0.01.

Previous studies have demonstrated that IL-11 can prevent the apoptosis and accelerate the recovery of small intestinal mucosa. PEDV-induced cell apoptosis plays a critical role in facilitating viral replication and pathogenesis. Upon IL-11 treatment, PEDV-induced apoptosis was quantitatively evaluated using Annexin V/PI flow cytometry. As shown in Figure 6A, treatment with IL-11 was able to notably decrease the percentage of early apoptotic cells induced by PEDV infection (lower right panel), which ranged from 9.78 to 11.9% during the course of PEDV infection.

Next, we investigated whether the anti-apoptotic function induced by pIL-11 is mediated through STAT3 signaling. IL-11 stimulation elicits the high expression of anti-apoptotic genes, such as Bcl-2, MCL-1 and Bcl-W, which are downstream of the IL-11/STAT3 signaling pathway [25]. Pre-stimulation with pIL-11 can reverse the changes in apoptotic related genes, following PEDV infection. Moreover, inhibition of STAT3 phosphorylation by S3I-201 almost completely abrogates the apoptosis related gene regulation and apoptosis inhibition function by pIL-11 (Figures 6A–C). These phenomena indicate that the anti-apoptosis effect induced by pIL-11 proceeds through the STAT3 signaling pathway. Combined with the results above, the presence of S3I-201 completely abolishes the antiviral activity of pIL-11 against PEDV (Figure 5C). The data presented here indicate that pIL-11 blocks PEDV-induced apoptosis via stimulation of STAT3 signaling, which then inhibits viral replication.

## Discussion

PEDV is the leading cause of piglet diarrhea worldwide and can lead to severe intestinal damage and significant mortality [26]. During PEDV invasion, the intestinal mucosal barrier can secrete a variety of cytokines and chemokines in response to viral infection [27]. Herein, we present evidence that IL-11 is significantly upregulated in Vero E6 cells during PEDV infection, and that the production of IL-11 is positively correlated with PEDV mRNA levels. Moreover, a similar IL-11 expression pattern was also observed in the intestine of piglets after PEDV inoculation. Previous studies have demonstrated that IL-11 can play a protective role, through anti-apoptosis, proliferation promotion, nutrition and downregulation of pro-inflammatory cytokines, in intestinal epithelial cell damage caused by various factors [28–30]. Therefore, we further investigated the biological function of PEDV-induced IL-11 expression.

Next, pIL-11 protein was produced and purified using an in vitro expression system, which indicates an obvious inhibition effect on PEDV in a dose-dependent manner. On the contrary, IL-11 KD Vero E6 cells exhibit significantly higher viral replication levels, compared with that of NC cells. In the intestinal mucosal immune system, the synergetic feedback loop among cytokines, including IL-22, IL-18 and IFN-γ, consists of a complex mutual regulating cytokine network [31, 32]. These cytokines can provide synergistic innate immunity to curtail viral infections, especially during early life stages, when components of the adaptive immune system are not yet fully operative. Our study is the first to report on the notable and previously unappreciated role of IL-11 in protecting the intestine against viral infection, which broadens our knowledge of the function of IL-11. We hypothesize that pIL-11 may act as an important candidate in these synergistic cytokine networks. Furthermore, the combination of IL-11 with with IL-22 or IFN-γ may be a potential therapeutic strategy for efficient antiviral immunity against gut coronaviral infections.

The mechanism of pIL-11 antiviral activity is also demonstrated by our results. IL-11 induces three major signaling transduction pathways: Jak/STAT, ERK, and PI3K/Akt, of which the PI3K/Akt and ERK pathways play an important role in regulating the growth, migration, differentiation and apoptosis of intestinal epithelial cells [33, 34]. However, none of these two signalling pathways participate in the pIL-11-induced antiviral effect observed in the present study. Our study demonstrates that pIL-11 decrease PEDV replication accompanied with phosphorylation of STAT3, while STAT3 inhibitor could significantly reverse the inhibitory effect of pIL-11. These results indicate that pIL-11 activates the IL-11/STAT3 signaling pathway in Vero E6 cells, ultimately improving their antiviral action capacity. STAT3 plays an important role in IL-11 activation of the JAK/STAT signal transduction pathway that regulates the expression of downstream target genes involved in promoting the repair of injured intestinal mucosal cells under pathological conditions [35, 36]. For example, IL-11 upregulates survivin expression in endothelial cells by activating STAT3 and inhibiting apoptosis [37]. Moreover, IL-11 also protects against ischemic intestinal epithelial injury by activating the JAK/STAT3 signaling pathway, upregulation of Bcl-2, PCNA, as well as the downregulation of Bax [38].

In this study, we found that pIL-11 is able to activate the JAK/STAT3 pathway and upregulate anti-apoptotic genes, which play a protective role against PEDV-induced apoptosis. Previous research has shown that the cytopathology of PEDV infection, represented by vacuolation and syncytia formation in vitro, is associated with the apoptotic process. Apoptosis induced by PEDV enhances the release and dissemination of viral progeny for further invasion, which facilitates viral replication and pathogenesis. Hence, we suggest that IL-11 may be able to regulate the expression of apoptosis-related genes and inhibit apoptosis by stimulating the JAK-STAT3 pathway, leading to inhibition of viral replication and production.

Our study demonstrates the antiviral activity of IL-11, which is positively correlated with PEDV infection in vitro and in piglets. Furthermore, the antiviral activity of pIL-11 proceeds via the activation of STAT3 signaling and enhances the inhibition of epithelial cell apoptosis. We conclude that IL-11 may be a potential therapeutic target against PEDV infection in piglets. However, further studies are required to better understand the complex IL-11-related regulatory mechanisms of the antiviral host response during viral infection. In addition, the potential antiviral function of IL-11 in combating other intestinal viruses, including TGEV, porcine rotavirus (PoRV) and Porcine deltacoronavirus (PDCoV), associated with diarrhea in suckling piglets is worthy of further research.

## Supplementary information

**Additional file 1. shRNA targeting sequences against IL-11.**

**Additional file 2. Standard curve for IL-11 (A) and PEDV M gene (B).**

**Additional file 3. IL-11 knockdown efficiency was verified by ELISA.**

**Additional file 4. pIL-11 treatment and knockdown did not affect cell viability.** (A) Cell viability was determined by CCK-8 assay after treatment of the Vero E6 cells with different concentrations of pIL-11 for 18 h. (B) NC and IL-11 KD Vero E6 cells were plated and culture to 70% confluent monolayers for the CCK-8 assay.

**Additional file 5. Cell viability assay after different inhibitor treatments.** Cell viability was determined by a CCK-8 assay after treatment of the Vero E6 cells with different inhibitor concentrations including S3I-201 for 24 h (A), LY294002 and MK-2206 2HCl for 2 h (B).
