# Supplementary material for: Antiviral activity of interleukin-11 as a response to porcine epidemic diarrhea virus infection
Source: Vet Res. 2019 Dec 21;50:111. doi: 10.1186/s13567-019-0729-9 (PMC6925494; doi:10.1186/s13567-019-0729-9)
Supplement: Supplementary file 1 — Additional file 1. shRNA targeting sequences against IL-11. [file 13567_2019_729_MOESM1_ESM.docx]

**Additional file 1. shRNA targeting sequences against IL-11**

| **Genes** | **Primers** | **Sequence (5'-3')** |
| --- | --- | --- |
| ShRNA IL11-1 | Forward | GATCCGGATTCTTGTGTCCACAGACTCGAAAGTCTGTGGACACAAGAATCCTTTTTG |
|  | Reverse | AATTCAAAAAGGATTCTTGTGTCCACAGACTTTCGAGTCTGTGGACACAAGAATCCG |
| ShRNA IL11-2 | Forward | GATCCGCCTGGGCAGGAATACATACTCGAAAGTATGTATTCCTGCCCAGGCTTTTTG |
|  | Reverse | AATTCAAAAAGCCTGGGCAGGAATACATACTTTCGAGTATGTATTCCTGCCCAGGCG |
| ShRNA IL11-3 | Forward | GATCCACCTTTGTGAGACGAAGAACACGAATGTTCTTCGTCTCACAAAGGTTTTTG |
|  | Reverse | AATTCAAAAACCTTTGTGAGACGAAGAACATTCGTGTTCTTCGTCTCACAAAGGTG |
