# Supplementary figures and images for: Antiviral activity of interleukin-11 as a response to porcine epidemic diarrhea virus infection
Source: Vet Res. 2019 Dec 21;50:111. doi: 10.1186/s13567-019-0729-9 (PMC6925494; doi:10.1186/s13567-019-0729-9)

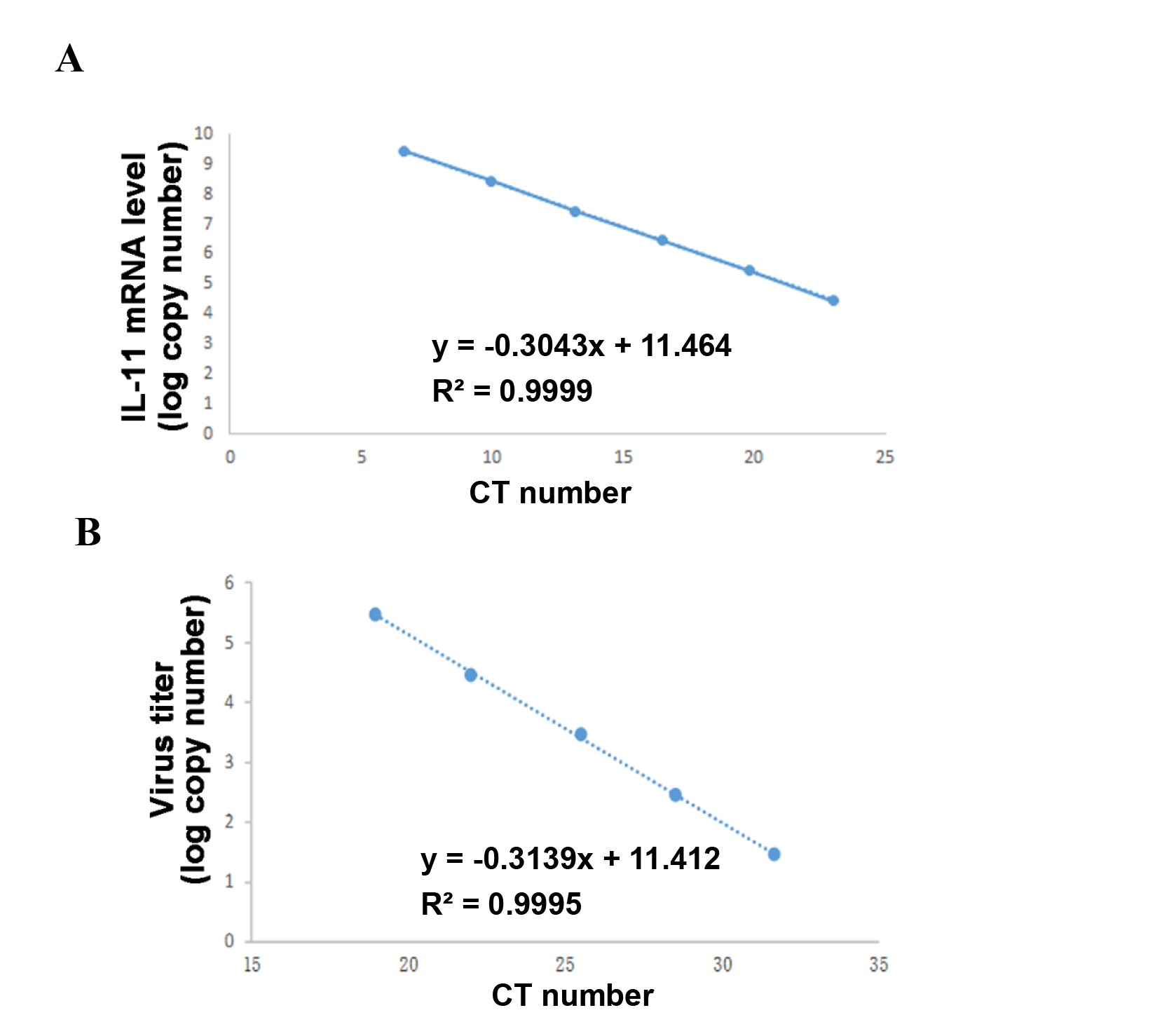

Supplement: Supplementary file 2 — Additional file 2. Standard curve for IL-11 (A) and PEDV M gene (B). [file 13567_2019_729_MOESM2_ESM.tif]

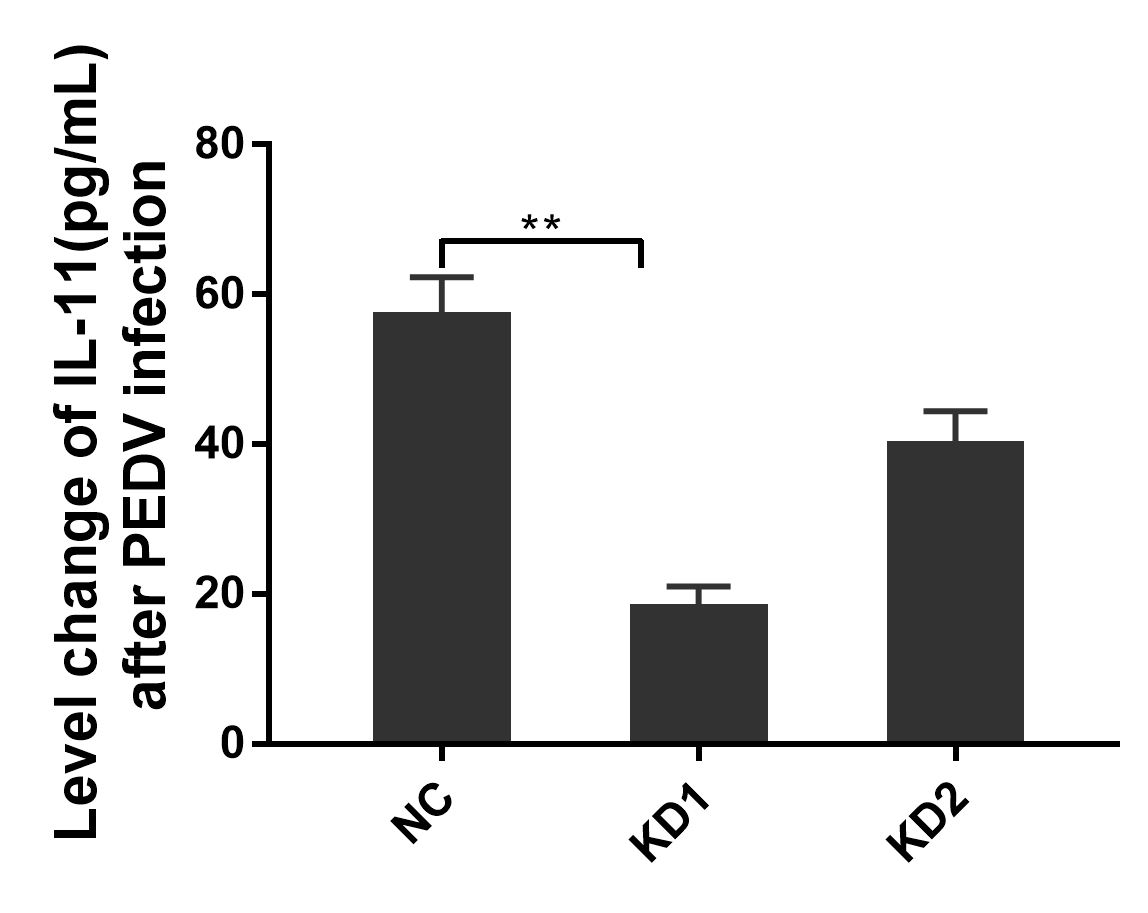

Supplement: Supplementary file 3 — Additional file 3. IL-11 knockdown efficiency was verified by ELISA. [file 13567_2019_729_MOESM3_ESM.tif]

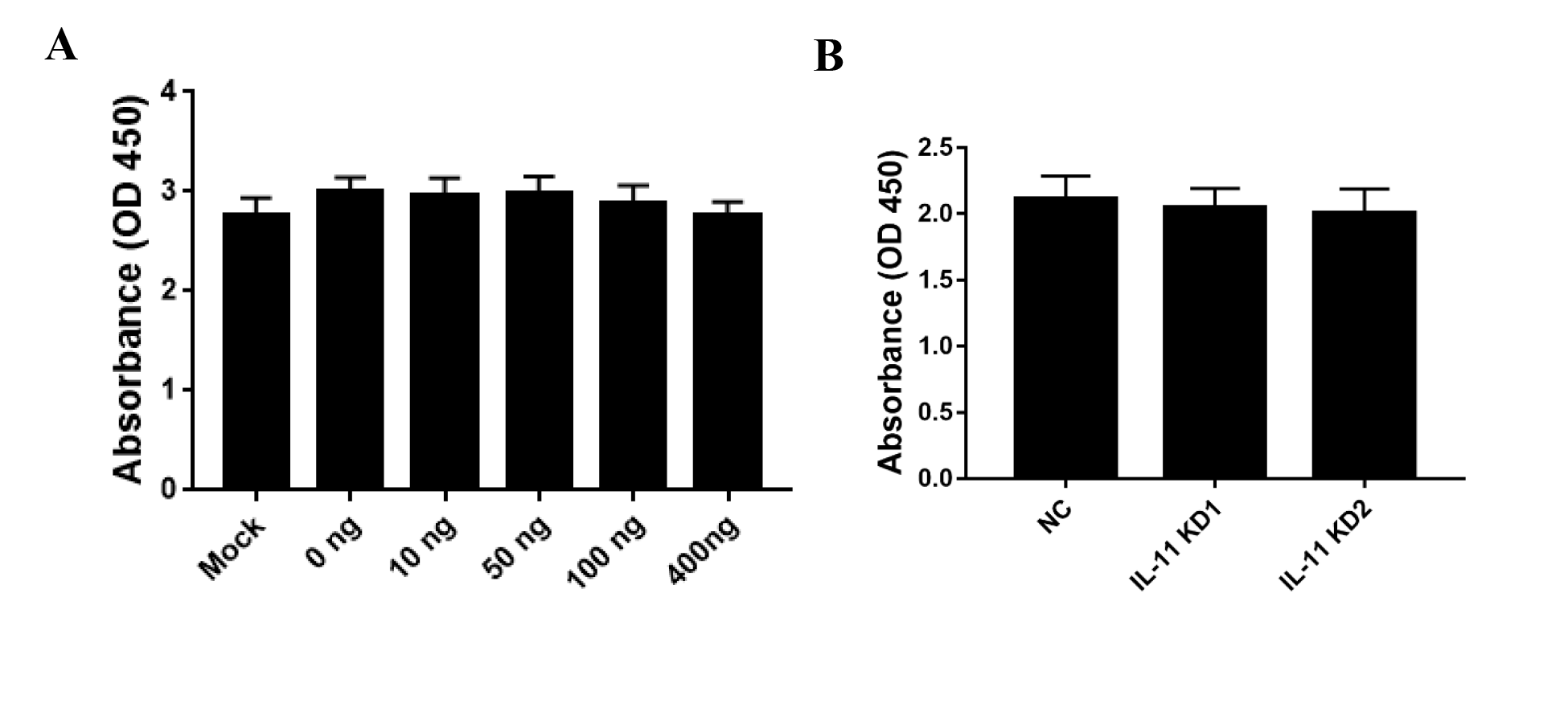

Supplement: Supplementary file 4 — Additional file 4. pIL-11 treatment and knockdown did not affect cell viability. (A) Cell viability was determined by CCK-8 assay after treatment of the Vero E6 cells with different concentrations of pIL-11 for 18 h. (B) NC and IL-11 KD Vero E6 cells were plated and culture to 70% confluent monolayers for the CCK-8 assay. [file 13567_2019_729_MOESM4_ESM.tif]

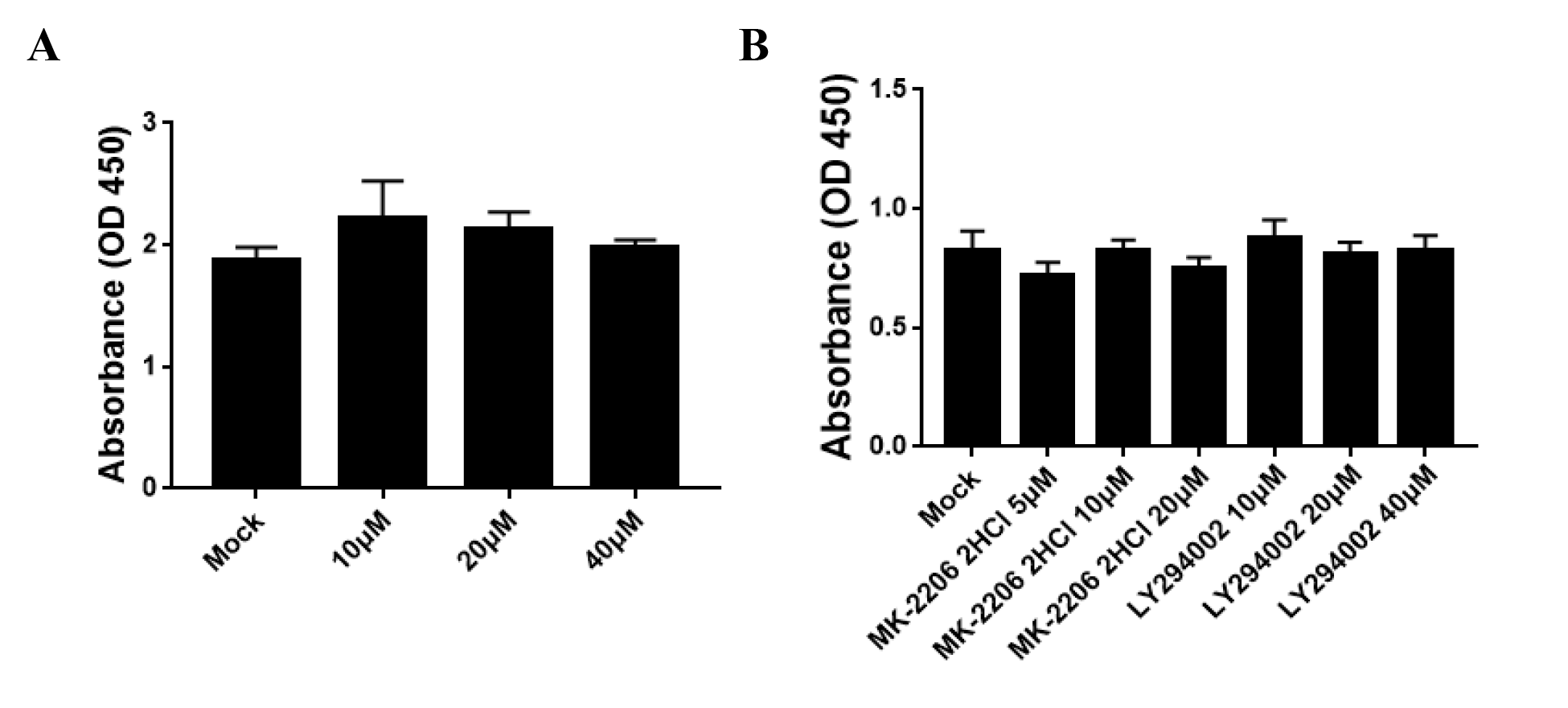

Supplement: Supplementary file 5 — Additional file 5. Cell viability assay after different inhibitor treatments. Cell viability was determined by a CCK-8 assay after treatment of the Vero E6 cells with different inhibitor concentrations including S3I-201 for 24 h (A), LY294002 and MK-2206 2HCl for 2 h (B). [file 13567_2019_729_MOESM5_ESM.tif]
